# United States Veterinary Medical Association

**Published:** 1895-01

**Authors:** 


### UNITED STATES VETERINARY MEDICAL ASSOCIATION.

THE following appointments of the United States Veterinary Medical Association have been made by the President, and acceptance on the part of the appointees has been received:

*President.*—W. Horace Hoskins, 3452–54 Ludlow Street, Philadelphia, Pa.

*Vice-Presidents.*—J. F. Winchester, Lawrence, Mass.; T. J. Turner, Columbia, Mo.; W. L. Williams, Bozeman, Mont.

*Secretary.*—Leonard Pearson, 2024 Pine Street, Philadelphia, Pa.

*Treasurer.*—Jas. L. Robertson, 409 Ninth Avenue, New York City.

*Comitia Minora.*—W. Horace Hoskins, *President*, 3452–54 Ludlow Street, Philadelphia, Pa.; J. F. Winchester, *Vice President*, Lawrence, Mass.; T. J. Turner, *Vice-President*, Columbia, Mo.; W. L. Williams, *Vice-President*, Bozeman, Mont.; Leonard Pearson, *Secretary*, 2024 Pine Street, Philadelphia, Pa.; Jas. L. Robertson, *Treasurer*, 409 Ninth Avenue, New York City, *ex officio*. A. S. Clement, *Chairman*, 916 Cathedral Street, Baltimore, Md., Thomas B. Rayner, Chestnut Hill, Philadelphia, Pa.; F. H. Osgood, 50 Village Street, Boston, Mass.; Olof Schwarzkopf, 1629 Wabash Avenue, Chicago, Ill.; R. S. Huidekoper, 155 W. Fifty-sixth Street, New York City.

*Committee on Intelligence and Education.*—C. A. Cary, *Chairman*, Auburn, Ala; W. B. Niles, Ames, Iowa; Geo. C. Faville, 46 Charlotte Street, Norfolk, Va.; R. A. Archibald, Sacramento, Cal.

*Finance Committee.*—Wm. Dougherty, *Chairman*, 1035 Cathedral Street,

Baltimore, Md.; D. J. Dixon, 366 Washington Street, Hoboken, N. J.; L. H. Howard, 1440 Washington Street, Boston, Mass.

*Committee on Diseases.*—M. R. Trumbower, *Chairman*, 502 Third Avenue, Sterling, Ill.; M. H. Reynolds, St. Anthony Park, Minn.; S. J. J. Harger, 3608 Pine Street, Philadelphia, Pa.; John Faust, 209 Union Street, Poughkeepsie, N. Y.; R. R. Dinwiddie, Fayetteville, Ark.

*Prize Committee.*—Tait Butler, *Chairman*, Agricultural College, Miss.; L. McLean, 14 and 16 Nevins Street, Brooklyn, N. Y.

*Army Legislation.*—J. P. Turner, *Chairman*, Fórt Myer, Va.; W. B. E. Miller, 527 Penn Street, Camden, N. J.; Jas. A. Waugh, 101 North Avenue, Allegheny, Pa.

*Publication Committee.*—A. T. Sellers, *Chairman*, 444 Benson Street, Camden, N. J.; Leonard Pearson, 2024 Pine Street, Philadelphia, Pa.; Jas. B. Rayner, West Chester, Pa.

*Special Committees: Act of Incorporation.*—Wm. Dougherty, *Chairman*, 1035 Cathedral Street, Baltimore, Md.; J. C. McNeil, 26 Fourth Street, Pittsburg, Pa.; F. S. Allen, 800 North Seventeenth Street, Philadelphia, Pa.

*Revision of Constitution and By-Laws.*—W. Horace Hoskins, *President*, 3452–54 Ludlow Street, Philadelphia, Pa.; J. F. Winchester, *Vice-President*, Lawrence, Mass.; T. J. Turner, *Vice-President*, Columbia, Mo.; W. L. Williams, *Vice-President*, Bozeman, Mont.; Leonard Pearson, *Secretary*, 2024 Pine Street, Philadelphia, Pa.; Jas. L. Robertson, *Treasurer*, 409 Ninth Avenue, New York City, *ex-officio*. W. B. E. Miller, *Chairman*, 527 Penn Street, Camden, N. J.; S. Stewart, 7½ St. James Street, Kansas City, Kans.; John M. Parker, 24 Essex Street, Haverhill, Mass.

*Resident State Secretaries.*—C. A. Cary, Auburn, Ala.; R. R. Dinwiddie, Fayetteville, Ark.; J. K. Thompson, Pueblo, Col.; H. P. Eves, 507 West Ninth Street, Wilmington, Del.; F. H. P. Edwards, Iowa City, Iowa; R. H. Harrison, Atchison, Kans.; Jas. B. Paige, Amherst, Mass.; R. A. Ramsey, Mexico, Mo.; W. L. Williams, Bozeman, Mont.; H. J. McClellan, Lansdowne, Pa.

